# Development, launch, and scale-up of health products in low-income and middle-income countries: a retrospective analysis on 59 health products

**DOI:** 10.1016/S2214-109X(25)00062-2

**Published:** 2025-05-21

**Authors:** Wenhui Mao, Elina Urli Hodges, Armand Zimmerman, Ernesto J Ortiz, Kelly Kilburn, Diana Silimperi, Krishna Udayakumar

**Affiliations:** a Duke Global Health Innovation Center, Duke Global Health Institute, Duke University, Durham, NC, USA; b Innovations in Healthcare, Duke University, Durham, NC, USA

## Abstract

**Background:**

Low-income and middle-income countries (LMICs) bear a heavy burden from communicable, maternal, and childhood diseases, yet access to health products addressing these burdens is lagging. We aimed to provide empirical evidence about the timelines, and influencing factors, of the end-to-end scale-up pathway of health products in LMICs.

**Methods:**

We identified products that met our prespecified inclusion criteria. We documented the dates and intervals for five key milestones in the pathway: ideation, proof of concept, regulatory approval, first LMIC country launch, and at least 20% uptake across LMICs. We then explored the association between pace of this pathway and ten characteristics, such as product types, buying environment, and market type. We used robust regression models to test our hypothesis that multiple factors affect a product's pathway to scale-up in LMICs.

**Findings:**

Among 59 included products, the median time to bring a product from ideation to at least 20% uptake in LMICs was 13·5 years (IQR 6·8–41·6). Ideation to proof of concept was the longest interval (median 6·9 years) for most product types, followed by the first country launch to at least 20% uptake in LMICs (median 3·8 years). Infectious disease products tended to need the longest time to launch, whereas maternal and child health products took the longest to scale up. Products with consumer or institutional markets launched and scaled up faster than those with global markets by 6 years and 2 years, respectively (p=0·012 and p<0·0001). Other factors, such as type of developer and buying environment, also had an effect on launch and scale-up timelines.

**Interpretation:**

Stakeholders should make long-term plans to introduce and scale up new products. Better coordination and planning across different stages and among different players could accelerate the process.

**Funding:**

Bill & Melinda Gates Foundation.

## Introduction

In 2021, the mortality rate from communicable, maternal, neonatal, and nutritional diseases in low-income countries was 3·2 times higher than the average across high-income countries.^1^ Although a multitude of health products exist to address these diseases, scale-up of such products in countries where these diseases are most burdensome is lagging.^2^ For example, the 2024 WHO malaria report found a substantial and growing coverage gap of malaria products.^3^ Enhanced and targeted investments to scale up health products addressing infectious, child, and maternal mortality could avert 10 million deaths by 2035, with economic benefits exceeding the cost of investment by a factor of 9 to 20.^4^ Ensuring that such investments are optimally disbursed to maximise impact requires an understanding of key factors that hinder or accelerate the launch and scale-up of health products to reach those who need them most.

A review reported an average of 10·5 years in the USA for a drug to progress from phase 1 development to regulatory approval,^5^ and the majority of drugs made very modest progress to uptake during their first 5 years on the market.^6^ Similarly, the same process took an average of 10 to 15 years in Europe.^7^ However, there is limited information about the timeline for launch and scale-up in low-income and middle-income countries (LMICs). Qualitative analyses, largely based on case studies, have identified that advocacy, resources, political will, supply chains, health-system governance, product characteristics, training, monitoring and evaluation, and collaboration are key elements influencing the success of scale-up.^8^ However, few studies, if any, have been justified with empirical evidence about the effect of such factors on the full process from ideation through to scale-up.

To address the evidence gap, we aimed to answer the research questions: how long did a health product take from ideation to scale-up and what are the enabling factors and barriers associated with each stage and the pathway to scale-up as a whole? We hypothesised that multiple factors affect a product's pathway to scale-up in LMICs.

**Research in context**

**Evidence before this study**

Although a number of health products, such as drugs, devices, vaccines, and diagnostics, have been developed, the launch and scale up of such health products is lagging, particularly in low-income and middle-income countries (LMICs). To better understand the end-to-end pathway of the launch and scale up of health products, we searched PubMed from Dec 5 to Dec 19, 2022, for articles published in English with the following search terms: (“research and develop*” OR “regulatory” OR “launch” OR “scale” OR “implement”) AND (“accelerat*” OR “delay” OR “enabl*”OR “barrier” OR“sustain*”) AND (“product” OR “innovation” OR “drug” OR “device” OR “vaccine” OR “diagnostic”) AND “health”. We found mostly qualitative studies or case studies that discuss the factors that might affect the end-to-end pathway of the launch and scale up of health products. Quantitative evidence has been found about the success rate and timeline of research and development, mostly in high-income countries. We found limited data on the timeline and influencing factors for launch and scale up in LMICs. We also observed that research and development, launch, and sustainable scale up were studied separately instead of as a connected process.

**Added value of this study**

This study is the first to report on the timeline from ideation to sustainable scale up of health products across different product types, health conditions, and in LMICs. It is also the first study to provide empirical evidence on the effect of different factors on the end-to-end pathway of the launch and scale up of health products in LMICs. We found that among 59 included health products, the median time to bring a product from ideation to at least 20% uptake in LMICs was 13·5 years. Ideation to proof of concept was the longest interval (median 6·9 years) for most product types, followed by the first country launch to at least 20% uptake in LMICs (3·8 years). Multiple factors, such as type of developer, market type, and buying environment, were observed to have an impact on launch and scale timelines.

**Implications of all the available evidence**

Funders, developers, regulators, and country policy makers can use the findings from this Article to set more realistic expectations, create enabling environments, and more efficiently direct resources to support the development, launch, and scale up of health products to reach more people. Better coordination and planning across different stages along the development to scale up pathway, and among different players and stakeholders, could accelerate the process. A crucial area for future investment would be the establishment of a publicly available evidence base to prospectively track the end-to-end launch and scale up of health interventions, with a standardised set of global and country level milestones and characteristics.

## Methods

### Conceptual framework, measurement, and selection of health products

We adapted the Scaling Innovation framework by the International Development Innovation Alliance to develop milestones according to the different stages of product development and access, from ideation through to sustainable scale-up.^9^ We define launch as the time between ideation to first country launch and scale as the time between the first country launch to LMIC uptake of at least 20%. To measure these stages, data on five milestones were collected for each product: ideation, proof of concept, first regulatory approval, first LMIC country launch, and LMIC uptake of at least 20%. Informed by the framework and other studies,^10^, ^11^ we selected the following ten characteristics of products based on the clarity of their definitions and the availability of reliable information: health condition; applicability to health or care pathway (type of care); type of developer; whether the product has a champion (ie, products or whether development and procurement were led or championed by prominent global organisations); buying environment (ie, centralised buying environments are those in which approximately 80% or more of the product is procured by one or several large buyers, eg, centralised—long-lasting insecticidal nets are mostly procured through large global buyers such as The Global Fund to Fight AIDS, Tuberculosis and Malaria); whether the product is developed specifically for LMICs; competition (substantial product competition such as multiple generic versions of the product or substantial competition among brands); whether the product requires targeting to specific sub-populations to be cost-effective; main market type (such as a globally coordinated market, local institutional market, or consumer market); and target channel (eg, public is when approximately 80% or more of the product is targeted to public channels as opposed to private pharmacies and facilities. Private is when approximately 80% or more of the product is targeted to private pharmacies and facilities as opposed to public channels. Mixed channels have more distribution across public and private facilities; appendix pp 1–2; full methods have been published elsewhere).^12^

There is no agreed list on prioritised health products for LMICs. Therefore, the selection of products in this study is exploratory, aiming to cover diverse products and explore the publicly available information. The products were identified through several steps: (1) an initial list, identifying potential products from multiple sources, such as PATH's Innovation Countdown 2030 report,^13^ *Saving Lives at Birth: A Grand Challenge for Development*,^14^ WHO and Partnership for Maternal, Newborn, and Child Health,^15^ and the Global Health Technologies Coalition; (2) pilot data collection on 12 diverse products regarding health condition, product type, and stage of implementation to explore the availability and quality of data (appendix pp 3–6); and (3) inputs from an advisory board^16^ consisting of policy makers, global health programme implementers, researchers, funders, and developers to both snowball expansion of the product list and to select the products to be included in this analysis. The 59 selected products cover diverse product categories, addressing diverse health conditions and, cross different eras of development (panel; appendix pp 3–6).

Panel **Included health products**

Among the 59 included health products, there are 21 vaccines, 18 drugs (including supplements), eight devices, seven diagnostics, and five vector control products. Below we list the products by product type and health conditions.

**Vaccines**

We included: 13 COVID-19 vaccines (Bharat Biotech COVAXIN; CanSino Ad5-nCoV; CureVac CVnCoV; Gamaleya Sputnik V; Janssen (J&J) Ad26.COV2.S; Moderna Spikevax; Novavax NVX-CoV2373; Oxford-AstraZeneca AZD1222 AZ; Oxford-AstraZeneca AZD1222 SKB; Pfizer–BioNTech BNT162; SII Covishield; Sinopharm SARS-CoV-2; and Sinovac Coronavac); seven non-COVID-19 vaccines for infectious diseases (Bivalent Oral Polio Vaccine, Ervebo, Gardasil, Japanese Encephalitis Vaccine, MenAfriVac, Novel Oral Polio Vaccine type 2, and Rotavac); and one non-COVID-19 vaccine for maternal and child health condition (RotaTeq).

**Drugs**

We included: ten drugs for infectious diseases (artesunate injection, rectal artesunate, coartem, coartem dispersible, dapivirine vaginal ring, pre-exposure prophylaxis, pretomanid, pyramax, pyramax granules, and tafenoquine); seven drugs and supplements for maternal and child health conditions (antenatal corticosteroids, chlorhexidine, magnesium sulfate, oral rehydration solution, pediatric tuberculosis medicines, tranexamic acid, and vitamin a); and one drug for neglected tropical diseases (fexinidazole).

**Devices**

We included: six devices for maternal and child health conditions (Bilichek, Bubble Continuous Positive Airway Pressure, ESM-Uterine Balloon Tamponade, MiracleFeet Brace, Moyo Fetal Heart Rate Monitor, and Sayana Press); one for infectious disease (Pratt Pouch); and another for neglected tropical disease (Tiny Targets).

**Diagnostics**

We included: six diagnostics for infectious diseases (Bioline Malaria, HIV self-test, Oraquick HIV self-test, SD Bioline Duo Rapid test, Xpert HIV-1 Assay, and Xpert MTB/RIF); and one for maternal and child health conditions (Congo Red Dot Paper Test).

**Vector control**

All included vector control products are for infectious diseases: Cielo ULV, long-lasting insecticidal nets, Royal Sentry 2.0, SumiShield, and Tsara.

### Data collection

We collected the dates for five milestones through desk review and outreach to key stakeholders (appendix p 2). We developed an operational protocol and a data entry form to collect the data (including the source or link, type of source, and quality).^14^ We started the search process for each product by understanding general information, including the developer, characteristics of the product, market information, etc. Then a combination of product name and indicators was used to perform in-depth searches on manufacturer, developer, or partner websites, regulatory agency documents or websites, implementing organisation websites and reports, and academic journals. Cross-reference was used to source the information. Cross-reference and outreach to key stakeholders involved in the development and scaling up of the products were also used to obtain more information or referral on information sources.

Milestone dates were collected in the format day-month-year. For dates with only the year available, we assigned the milestone to be the midpoint of the year (ie, July 1st). For dates with the year and month available, but not the day, we assigned the milestone to be the midpoint of the month (ie, the 15th). LMIC coverage was measured from demand, supply, or policy perspectives (appendix pp 1–6). We selected the 20% uptake milestone due to the larger availability of data for this milestone. Additionally, we searched literature to collect ten qualitative characteristics such as buying environment and market type. Definitions and specific information about data collection can be found in the appendix (p 2). All data was collected by one researcher and verified by a second researcher. Disagreements were resolved through discussion between reviewers or through a group discussion.

When the interval between specific milestones of a product was not available (ie, one of the milestone dates was not found), we assigned the median of products of the same product type. If the median interval by product type was not available either, we then used the median interval of products with the same health condition. We made the following assumptions for products without certain characteristic information: if all other products of the same product type or the same health condition shared the same characteristic value, then this value was assigned to the missing datapoint (for example, all vector control products had a centralised buying environment, so a vector control product with a missing value for buying environment was assigned a centralised buying environment); and if one characteristic was associated with another characteristic, then these two characteristics were used to assign missing values (for example, all products with centralised buying environments also had global markets; therefore, products with a global market but that had a missing value for buying environment were assigned centralised buying environments). We also provided the analysis on the original data in the appendix (pp 8, 12).

### Data analysis

We took the difference between milestone dates to get the intervals, measured in years. We reported the median, IQR, and 25th and 75th percentiles across all products and disaggregated by product type and health condition. We used robust regression models with multiple explanatory variables to test our hypothesis on product characteristics and milestone intervals. Robust regression is selected for its better capacity than the ordinary least squares regression in estimating data with outliers or influential observations. It uses an iteratively reweighted least squares method to assign a weight to each data point.^17^, ^18^, ^19^ We chose two dependent variables: ideation to first country launch and first country launch to LMIC uptake of at least 20% to reflect the launch and scale-up of products. For independent variables, we used different combinations of the characteristics that we collected. We tested Tolerance and Variance Infection Factor and concluded there was no severe collinearity (Tolerance and Variance Infection Factor<10) in our main models. The main analysis was performed on all 59 products whereas the sub-analysis was performed on 46 non-COVID-19 products. Alternative models, including different model fittings and different combinations of variables, can be found in the appendix (pp 10–14). We report the beta coefficient, p value, and confidence interval (CI) at an alpha of 0·10 for all independent variables. Descriptive and regression analysis was performed in R (version 4.3.0) and StataSE 15 by StataCorp, respectively.

### Role of the funding source

The funder had no role in study design, data collection, data analysis, interpretation, or writing of the report.

## Results

**Figure 1:**
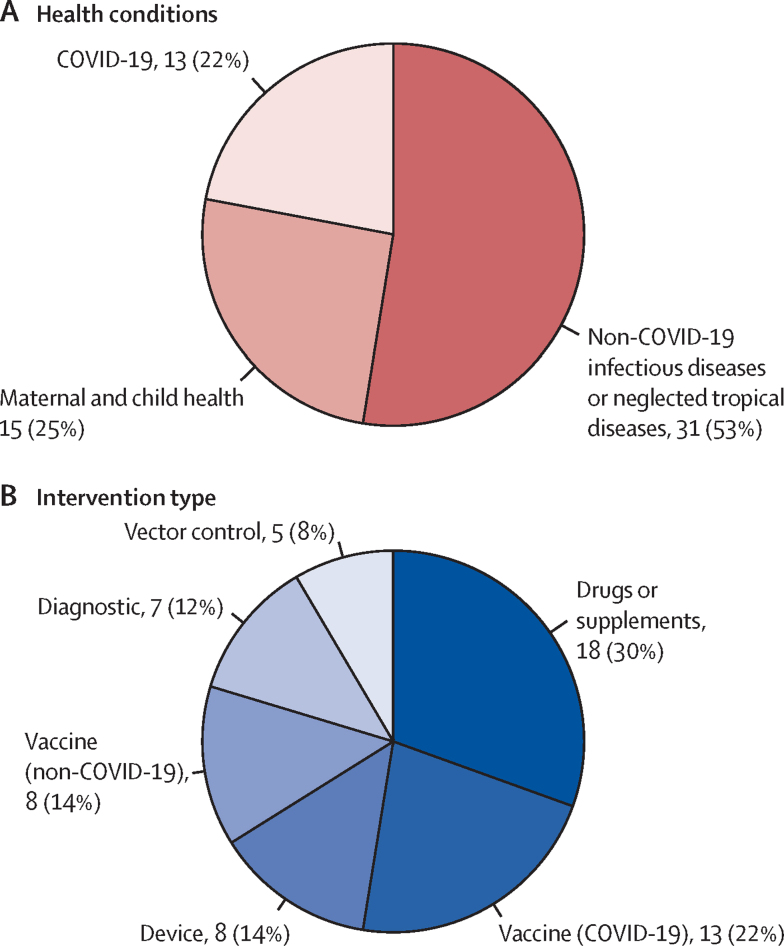
Product characteristics

Among 59 included products, 31 (53%) were for infectious diseases or neglected tropical diseases, 15 (25%) for maternal and child health conditions, and 13 (22%) for COVID-19 (figure 1). 39 (66%) were vaccines and drugs, followed by devices, diagnostics, and vector control products (figure 1; appendix pp 3–6, 8–9). Among all 16 variables for 59 products included in this study, missing values occurred at 148 of 944 total data points (15·7%); we recorded all the missing data, and our imputation approaches in the appendix (p 7).

Across all products, the median time to bring a product from ideation to at least 20% uptake in LMICs was 13·5 years (IQR 6·8–41·6; table 1). Specifically, the median time taken to launch was 9·0 years (1·7–14·8) and 3·8 years (0·7–21·0) to scale-up. During the launch process, ideation to proof of concept took the longest (6·9 years [1·6–16·1]), followed by another 2·2 years (0·14–4·2) to get regulatory approval (table 1). However, if we exclude COVID-19 products, these processes took much longer; specifically, the median time from ideation to at least 20% uptake in LMICs was 17·5 years (13·1–41·6). Additional timelines for products with data indicating further scale-up beyond 20% are presented in the appendix (p 15).

**Table 1:** Time between milestones (in years)

		**Median**	**IQR**
**All interventions (N=59)**			
Launch: ideation to first country launch		9·0	1·7–14·8
	Ideation to proof of concept	6·9	1·6–16·1
	Proof of concept to first regulatory approval	2·19	0·14–4·22
	First regulatory approval to first country launch	0·32	0·04–0·61
Scale: first country launch to LMIC uptake of at least 20%		3·8	0·7–21·0
Total: ideation to LMIC uptake of at least 20%		13·5	6·8–41·6
**All non-COVID-19 interventions (n=46)**			
Launch: ideation to first country launch		13·0	6·7–14·8
	Ideation to proof of concept	10·6	5·1–17·5
	Proof of concept to first regulatory approval	2·62	0·56–4·41
	First regulatory approval to first country launch	0·32	0·15–0·98
Scaled: first country launch to LMIC uptake of at least 20%		5·0	3·5–21·0
Total: ideation to LMIC uptake of at least 20%		17·5	13·1–41·6

**Figure 2:**
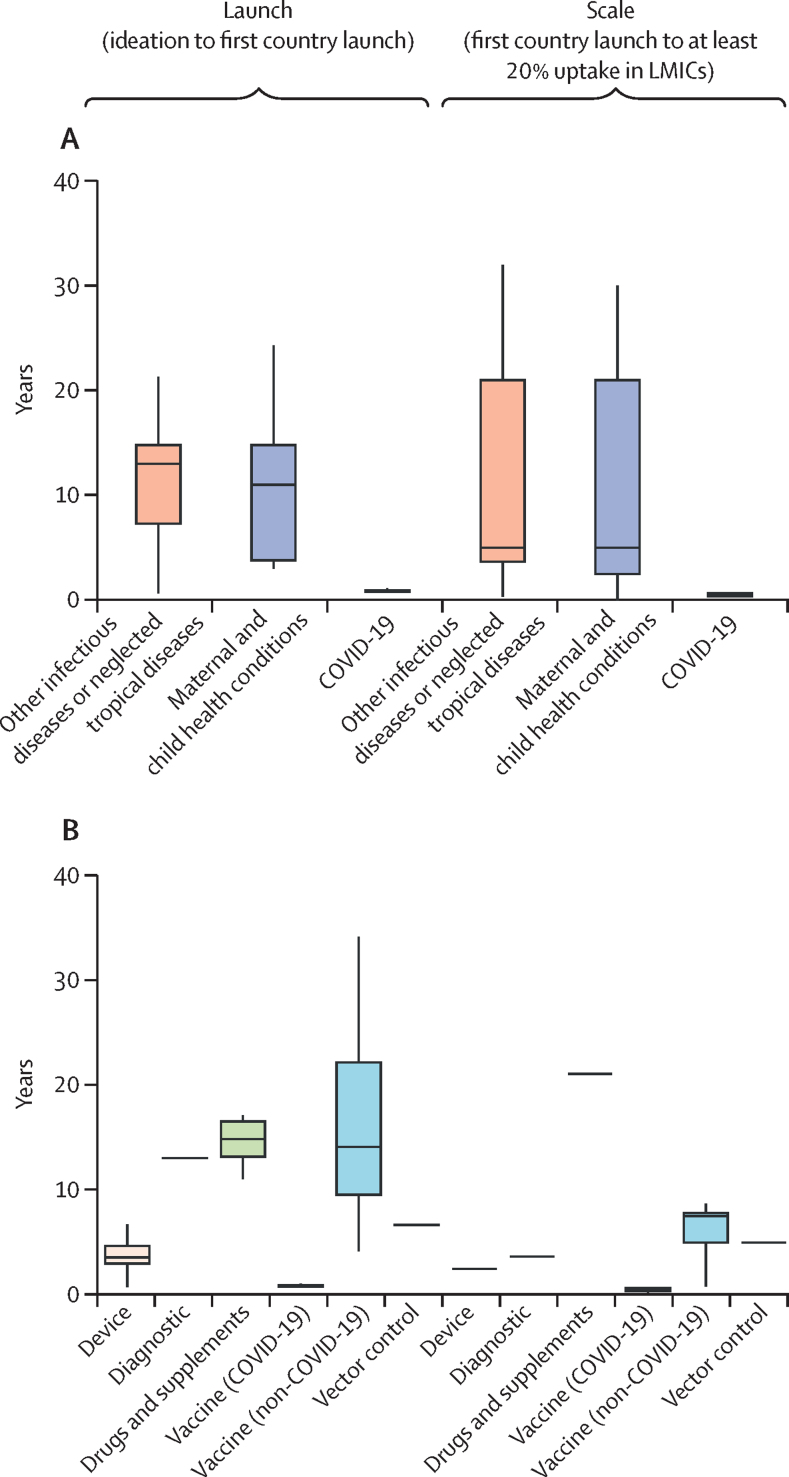
Milestone durations of different products (A) By health conditions. (B) By intervention type. Boxes show the middle 50% of the data (the IQR) and the whiskers show the range of the remaining data. LMICs=low-income and middle-income countries.

Among products for different health conditions, infectious diseases or neglected tropical diseases products tended to need the longest time to launch, whereas maternal and child health products took the longest to scale up (figure 2). Among different types of products, non-COVID-19 vaccines tended to need the longest time for both launch and scale-up whereas COVID-19 vaccines took the shortest duration (figure 2). Ideation to proof of concept appeared to be the longest interval for all product types except for drugs and supplements, which had the longest interval between first country launch to LMIC uptake of at least 20% (figure 2).

Products featuring different characteristics also took different times to launch and scale-up (table 2). Generally, products developed for certain market types had the largest variance to launch: products for consumer or institutional markets only took 3·0 years (IQR 0·9–13·0) to launch, whereas products for the global market took 13·5 years (6·7–16·5) to launch. Products with different market types had the largest variance to scale up. However, heterogeneity in interval to launch or to scale-up was large, even among products with the same characteristics.

**Table 2:** Time between key milestones (in years) by characteristics

	**All interventions (n=59)**		**All non-COVID-19 interventions (n=46)**	
	Launch (ideation to first country launch)	Scale up (first country launch to at least 20% uptake in LMICs)	Launch (ideation to first country launch)	Scale up (first country launch to at least 20% uptake in LMICs)
**Type of care**				
Prevention (n=34)	6·7 (0·9–14·3)	3·1 (0·7–7·5)	11·0 (6·7–20·2)	5·0 (3·8–14·8)
Diagnosis or screening (n=8)	13·0 (3·2–13·0)	3·7 (2·6–3·7)	13·0 (3·1–13·0)	3·7 (2·6–3·7)
Treatment (n=17)	14·8 (5·7–14·8)	21·0 (3·7–21·0)	14·8 (5·7–14·8)	21·0 (3·7–21·0)
**Developer**				
Private company (n=32)	6·7 (1·1–14·1)	4·1 (1·8–7·5)	11·0 (4·5–14·8)	5·0 (3·7–14·8)
Public–private collaboration (n=17)	12·6 (0·9–15·9)	3·7 (0·7–21·0)	14·8 (12·6–21·3)	21·0 (3·7–21·0)
Academic-non-governmental organisation (n=6)	5·4 (3·4–14·5)	2·5 (1·9–4·0)	5·4 (3·4–14·5)	2·5 (1·9–4·0)
**Champion**				
Clear champion (n=33)	6·9 (0·9–14·5)	3·7 (0·7–14·3)	13·0 (7·4–18·0)	6·4 (3·7–21·0)
No clear champion (n=26)	13·0 (3·9–14·8)	5·0 (2·5–21·0)	13·0 (5·3–14·8)	5·0 (2·5–21·0)
**Buying environment**				
Centralised (n=32)	10·3 (5·2–14·8)	5·0 (2·6–21·0)	12·0 (6·7–15·4)	5·0 (3·5–21·0)
Decentralised (n=27)	3·6 (0·9–14·8)	2·5 (0·7–21·0)	13·0 (4·7–14·8)	6·2 (2·8–21·0)
**Country-specific**				
LMIC-specific (n=23)	6·7 (3·0–13·0)	3·7 (2·5–6·4)	6·7 (3·8–13·0)	5·0 (2·5–7·0)
Not LMIC-specific (n=36)	13·0 (1·0–14·8)	4·1 (0·7–21·0)	14·8 (12·8–21·7)	21·0 (3·8–21·0)
**Product competition**				
No substantial competition (n=34)	4·5 (0·9–14·1)	2·5 (0·7–10·9)	12·8 (4·6–14·8)	7·0 (2·5–21·0)
Substantial competition (n=25)	13·0 (6·7–16·9)	5·0 (3·3–21·0)	13·0 (6·7–18·0)	5·0 (3·7–21·0)
**Targeting**				
Required (n=32)	5·7 (0·9–14·8)	3·1 (0·7–8·4)	14·2 (5·7–18·0)	5·0 (3·1–21·0)
Not required (n=27)	12·6 (4·2–14·8)	5·0 (2·5–21·0)	13·0 (6·7–14·8)	5·0 (3·3–21·0)
**Main market type**				
Consumer or institutional (n=31)	3·0 (0·9–13·0)	2·5 (0·7–5·0)	11·0 (3·6–14·8)	3·8 (2·5–21·0)
Global (n=28)	13·5 (6·7–16·5)	7·0 (3·7–21·0)	14·0 (6·7–17·1)	7·5 (3·7–21.0)
**Target channel**				
Mixed or private (n=20)	13·0 (3·9–14·8)	4·7 (2·6–21·0)	13·0 (4·8–14·8)	5·0 (3·0–21·0)
Public (n=39)	6·7 (0.9–14·9)	3·7 (0·7–8·7)	13·0 (6·7–21·3)	5·0 (3·7–21·0)

If a product had one or more of the following characteristics—for COVID-19 (compared with other infectious diseases), developed by a private company (compared with other types of developers), had a decentralised buying environment (compared with a centralised buying environment), were LMIC-specific (compared with non-LMIC-specific), for the consumer or institutional market (compared with for global market)—it had a substantially faster launch (table 3). If a product had one or more of following characteristics—for COVID-19, for diagnosis or screening (compared for prevention), had substantial product competition (compared for no substantial competition), and for the consumer or institutional market (compared for the global market), scale-up would have been substantially faster (table 3). For example, infectious disease and neglected tropical disease products took 12·4 years longer to launch and 4·3 years longer to scale up than COVID-19 products. Products specifically developed for LMICs launched 6·2 years faster than those not specifically for LMICs. And compared with products with global market, products with a consumer or institutional market launched 6·2 years faster and scaled up 2·3 years faster. Notably, several characteristics affected launch and scale-up in similar ways: compared with other infectious diseases, COVID-19 products had substantially shorter time for both the launch and scale-up, and products with consumer or institutional markets also had significantly shorter time for both launch and scale-up, than those with global markets. Some other factors affected launch and scale-up, but not all variables had significant effects on interval to launch, scale-up, or both (table 3). Regression models without COVID-19 products, with different model fitting, using original data, and with different combinations of control variables yielded similar results (appendix pp 10–14).

**Table 3:** Characteristics that affect launch and scale up

		**Model 1: launch (ideation to first country launch)**		**Model 2: scale up (first country launch to uptake of at least 20% in LMICs)**	
		Beta (p value)	90% CI	Beta (p value)	90% CI
Health condition: non-COVID-19 infectious diseases and neglected tropical diseases as reference					
	Maternal and child health and nutrition	0·5 (0·86)	−3·9 to 4·8	2·0* (<0·0001)	1·2 to 2·7
	COVID–19	−12·4* (<0·0001)	−17·2 to −7·7	−4·3* (<0·0001)	−5·2 to −3·5
Type of care: prevention as reference					
	Diagnosis or screening	−3·3 (0·27)	−8·2 to 1·6	−2·4* (<0·0001)	−3·2 to −1·5
	Treatment	−3·5 (0·14)	−7·3 to 0·4	15·1* (<0·0001)	14·4 to 15·7
Developer: private company as reference					
	Academic or non-governmental organisations	5·9† (0·053)	0·9 to 10·9	−0·5 (0·37)	−1·3 to 0·4
	Public–private collaboration	4·1‡ (0·046)	0·8 to 7·5	0·3 (0·44)	−0·3 to 0·9
Champion: clear champion as reference					
	No clear champion	−1·0 (0·61)	−4·2 to 2·2	−0·2 (0·55)	−0·8 to 0·4
Buying environment: decentralised as reference					
	Centralised	3·9† (0·099)	<0·0 to 7·8	0·6 (0·15)	−0·1 to 1·3
Country-specific: not LMIC-specific as reference					
	LMIC-specific	−6·2* (0·001)	−9·1 to −3·3	−0·3 (0·29)	−0·8 to 0·2
Product competition: no significant competition as reference					
	Significant product competition	2·2 (0·23)	−0·9 to 5·3	−1·0* (0·002)	−1·6 to −0·5
Targeting: requires targeting as reference					
	Does not require targeting	−1·3 (0·51)	−4·4 to 1·9	0·3 (0·45)	−0·3 to 0·8
Main market type: global market as reference					
	Consumer or institutional market	−6·2‡ (0·012)	−10·2 to −2·3	−2·3* (<0·0001)	−3·0 to −1·6
Target channel: public as reference					
	Private or mixed	0·3 (0·89)	−3·2 to 3·8	−0·1 (0·82)	−0·7 to 0·5
Constant		14·9	..	6·6	..
Model metrics					
	Observations	59	..	59	..

The regression analysis on a subset of interventions without COVID-19 vaccines is shown in the appendix (p 10). LMIC=low-income and middle-income country.

* p<0·01.

† p<0·1.

‡ p<0·05.

## Discussion

To the best of our knowledge, we present one of the first empirical studies to identify the time and influential factors of the end-to-end scale-up pathways for a broad set of health products. These findings can inform priorities and decisions for stakeholders, including product developers, funders, implementers who focus on certain stages of the end-to-end pathway, and communities of practice concentrated on specific diseases or health conditions, who can benefit from better understanding the time and key driving factors along the path to scale-up.

To launch a product and attain limited scale-up in LMICs can take more than a decade. Similar patterns have been reported in the USA and Europe, although scale-up data is sparse.^7^, ^8^, ^9^ These data highlight the urgency to address end-to-end development, introduction, and scale-up of health products in a more coordinated way. Our findings could inform funders and developers to set realistic expectations and allocate sufficient resources to launch and scale-up new products, including heightened emphasis on key barriers.^6^ Viewing product development through scale-up as an interconnected, non-linear process enables funders and developers to prepare adequate resources, partnerships, and handoffs to ensure products reach their intended users more effectively and efficiently. Such coordination could also reduce the total time to scale-up as funders and developers better prepare for and set the stage for implementation during the development stage.^20^ For example, a WHO report identified the need to address research, norms and standards, implementation, and advocacy as four strategic, interdependent areas to combat postpartum haemorrhage.^21^ Thus a more comprehensive and systematic approach should be considered beyond the product-by-product approach. Addressing complex health challenges requires integrated, bundled innovations that might include multiple health products (eg, diagnostics and therapeutics) along with new workforce and business models to unlock the full potential of innovations and enable sustainable scale-up.

We observed that certain characteristics influence the time needed for launch and scale-up, providing funders and developers with a roadmap to optimise planning for product development and ecosystem development. For example, while the needs to increase the research and development for maternal conditions has been well documented,^22^ we observed that only a limited number of products for maternal conditions have reached 20% of the target population. Specific contextual factors and barriers, including the lack of a centralised purchaser, the need to change the behaviour or preferences of end users, and the scarcity of skilled providers, need to be addressed comprehensively and simultaneously to enable health products to reach broader populations.

The massive investments and urgent partnerships in response to the COVID-19 pandemic highlighted opportunities to streamline and accelerate the pathway from development to implementation.^23^, ^24^ Investments by national regulatory agencies to achieve similar efficiency gains for other products during non-emergency conditions could substantially reduce the time needed to show proof of concept and therefore accelerate the launch of health products. Additionally, enhancing regulatory capacities and harmonisation at national, regional, and global levels can improve access to health products in LMICs.

Our analysis identified barriers and facilitators that could also be of value in decision making by investors, developers, and regulators. We found products developed by academic institutions, non-governmental organisations, or public–private partnerships are slower to launch than those developed by private companies, which aligns with other qualitative evidence.^10^ Our results also show that products with consumer or institutional markets and decentralised purchasing channels launch and scale up faster than products with global markets and centralised purchasing channels although previous evidence found mixed views.^25^, ^26^, ^27^ Although certain elements of both centralisation and decentralisation could be valuable to product procurement systems, our results support the notion that procurement at a national or local, rather than global level could lessen the time needed to launch products. Additional research is needed to confirm these findings. Downstream factors, such as product demand, distribution, and delivery, and the capacity of health systems, have played an increasing role in scaling up and should be better tracked and planned.

We highlight several limitations that have implications for future research. First, not all products available in LMICs have been included in this study. Most included products were launched in at least one LMIC. Considering the high rate of failure across product research and development (ie, 7·9% of all candidates from phase 1 trials got approved in the USA, and 7–45% in Europe),^7^, ^9^ learning from failed products might generate additional insights. Second, our analysis relied on publicly available data and measurable indicators. For instance, the sales or procurement information of health products is normally not publicly available. The missing data might affect the overall assessment on timeline and the factors associated with faster or slower timelines. Better data capture and public data sharing should be encouraged across different stakeholders such as manufactures and suppliers. Another example of a data limitation is the fact that we were only able to find five products that attained 80% scale-up, and less than half of the included products have information on 20% scale-up in LMICs (appendix p 15). This evidence gap needs to be addressed for better understanding of the barriers to scale up. A publicly accessible database that prospectively tracks the end-to-end scale-up of health products, with a standardised set of global and country milestones and characteristics, could provide robust evidence to reduce the time needed to bring new products to users. Finally, our study considered only individual products, thus the bundling or integration of products across the care continuum could be another area for further study. Even with these limitations, this study provides valuable evidence to inform decision making in the research, development, regulation, launch, and scale-up of health products.

### Contributors

### Data sharing

All data presented is available from the Launch and Scale Speedometer website.

## Declaration of interests
