# Supplementary material for: Development, launch, and scale-up of health products in low-income and middle-income countries: a retrospective analysis on 59 health products
Source: Lancet Glob Health. 2025 May 21;13(6):e1132–9. doi: 10.1016/S2214-109X(25)00062-2 (PMC12100461; doi:10.1016/S2214-109X(25)00062-2)
Supplement: Supplementary appendix [file mmc1.pdf]

THE LANCET
Global Health

Supplementary appendix

This appendix formed part of the original submission and has been peer reviewed. We post it as supplied by the authors.

Supplement to: Mao W, Hodges EU, Zimmerman A, et al. Development, launch, and scale-up of health products in low-income and middle-income countries: a retrospective analysis on 59 health products. *Lancet Glob Health* 2025; **13:** e1132–39.

Contents

Appendix 1: Conceptual framework and definitions ... 1

Table S1. Definitions for milestones and characteristics ... 2

Appendix 2: List of products ... 3

Table S2. List of included products ... 3

Appendix 3: Missing data ... 7

Table S3. Patterns of missing milestone data ... 7

Appendix 4: Descriptive statistics of original data (i.e. with missing values) ... 8

Table S4-1. Product characteristics ... 8

Table S4-2. Time between milestones (in years) ... 8

Appendix 5: Alternative regression models ... 10

Table S5-1. Robustness regression without COVID-19 vaccines ... 10

Table S5-2. Regression results using OLS models ... 11

Table S5-3. Robustness regression results with original data ... 12

Table S5-4. Robust regression models with alternative dependent variables: from ideation to first country launch ... 13

Table S5-5. Robust regression models with alternative dependent variables: from first country launch to global uptake at least 20% ... 14

Appendix 6: Timeline for scale-up (selected products) ... 15

Table S6. Products with scale up data and their scale up timeline ... 15

Appendix 1: Conceptual framework and definitions

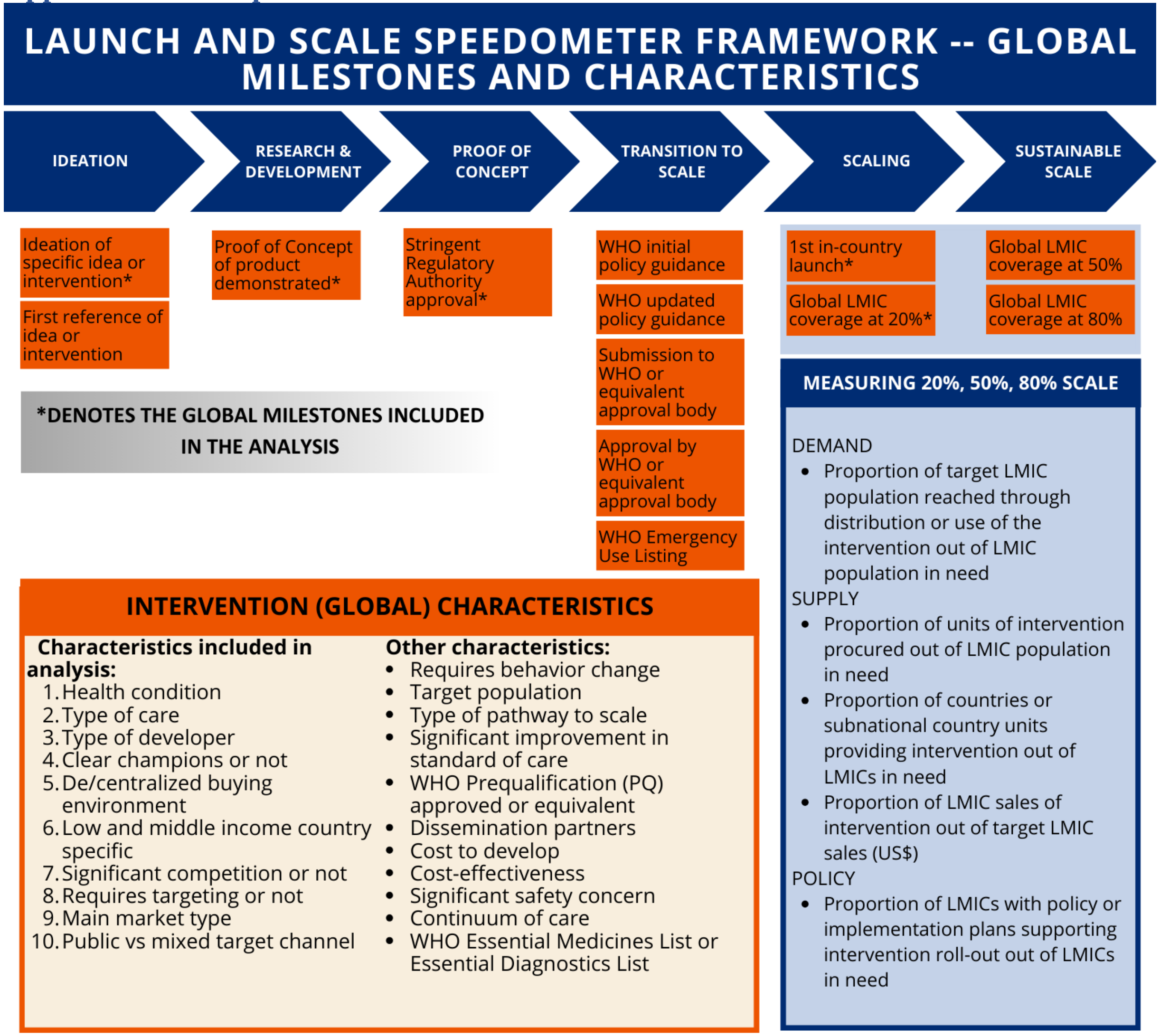

Note: for country milestones, please refer to Duke Global Health Innovation Center. Measuring and Analyzing the Launch and Scale of Life Saving Health Interventions: A User Guide. 2023 June. Available from: https://launchandscalefaster.org/sites/default/files/documents/User%20guide%202023_FINAL.pdf (Accessed June 30, 2023).

Table S1. Definitions for milestones and characteristics

Milestones /characteristics	Definition and measurement
Product Type	Categorical: drug, device, diagnostic, procedure, supplementation, vaccines
**FIVE MILESTONES**	
Ideation	Date of discovery or idea for the original product or product from which the current product is adapted. e.g. The first discovery of a treatment for malaria was artemisinin, which was isolated in 1972 by Chinese scientists from the sweet wormwood plant, Artemisia annua.
Proof of concept	Date product demonstrated to be safe and effective for intended purpose in humans, such as date results shared (e.g. publication) from clinical trials for drugs, diagnostics, and other products that require SRA approval (Phase II studies or equivalent). If no SRA approval necessary, then date that efficacy was demonstrated (randomized control trial (RCT) or equivalent published)
First regulatory approval	Date of first approval by a national regulatory authority
1st country launch	Date the product was used (launched / commercialized / procured) in a LMIC country for the first time outside of a research study
Low- and Middle-Income Countries (LMICs) uptake of the product at 20% *	Date that coverage of the product reached 20% globally using one of the global coverage indicators and an appropriate denominator that measured through:<br>1) Demand-side measures: the total LMIC population reached by the product (through its use) as a percentage of the total LMIC population with the health condition. Numerator: Total global population in LMICs reached by product through distribution or use of the product; Denominator: Global LMIC population with health issue or disease (in some situations can also be the addressable market or the unmet need, e.g. contraception)<br>2) Supply-side measures: the total units of product procured by LMICs as a percentage of the total LMIC population with the health condition. a) Numerator: Total number of units of product procured by LMICs; Denominator: Global LMIC population with health issue or disease* (in some situations can also be the addressable market or the unmet need, e.g. contraception); b) Numerator: Total # of LMIC countries providing the product (where it is available); Denominator: Total # of LMICs that have populations with health issue or disease; c) Numerator: Global sales of product in terms of value (US$) in LMICs; Denominator: Target global sales in terms of value (US$) in LMICs.<br>3) Policy measures: the total number of LMICs providing the product as a percentage of the total number of LMICs with populations that have the health condition. Numerator: Total # of LMICs with policy or implementation plans supporting roll-out of product; Denominator: Total # of LMICs that have populations with health issue or disease
**CHARACTERISTICS**	
Health condition	Categorical: Infectious disease, Neglected Tropical Diseases (NTDs), Maternal, Newborn and Child Health (MNCH), Nutrition
Continuum of care	Categorical: Prevention; Awareness; Screening; Diagnosis; Treatment; Monitoring/ After Care
Type of Developer	The type of organization where the idea and original development of the product came from. Categorical: For-profit company, non-profit company (NGO), academic institution, or other (specify)
Clear champion(s)	Clear champion(s): Products whose development and procurement were led/championed by prominent global organizations<br>e.g. Global health campaign initiated for product like for Sayana Press with multiple global family planning partners or the TB Alliance championed TB treatment.
Buying environment	Centralized buying environments are where ~80% or more of the product is procured by one or several large buyers (organizations / large governments)<br>e.g. Centralized—LLINs are mostly procured through large global buyers like Global Fund; Decentralized—Uterine balloon tamponades
LMIC specific	Low and middle-income country (LMIC) specific refers to products developed specifically for LMIC country use
Product competition	Significant product competition: Multiple generic versions of the product/ Significant competition among brands
Requires targeting	Products requiring targeting at specific sub-populations to be cost-effective<br>e.g. Products like Sayana Press that require targeting at specific sub-populations (mostly young women in need of modern contraceptive) to be cost-effective
Main market type	Globally Coordinated Market: "technologies such as vaccines are procured and financed through centralized channels."<br>Local Institutional Market: "national institutions, such as a ministry of health, purchase technologies such as drugs used for obstetric care, whether through their own resources or from donor grants or loans."<br>Consumer: "large number of disaggregated consumers buy health goods, such as oral rehydration salts or water filters, for their own use."<br>e.g. LLINS are global because they are procured and finalized through centralized channels. Chlorhexidine is institutional because national institutions (like Ministry of Health) purchase for newborn care. Sayana Press is a consumer market mainly because consumers purchase it for their own use.
Target channel	Public is where ~80% or more of product is targeted to public channels as opposed to private pharmacies and facilities. Private is where ~80% or more of product is targeted to public channels as opposed to private pharmacies and facilities. Mixed channels have more distribution across public and private facilities.<br>e.g. LLINS are public mostly, MiracleFeet is Private, and Sayana Press is mixed

Reference: Duke Global Health Innovation Center. Measuring and Analyzing the Launch and Scale of Life Saving Health Interventions: A User Guide. 2023 June. Available from: https://launchandscalefaster.org/sites/default/files/documents/User%20guide%202023_FINAL.pdf (Accessed June 30, 2023).

Appendix 2: List of products

Table S2. List of included products

Product	Commercial name	Type	Health condition	Continuum of care	Specific diseases	Pilot#	Scale up data
Bharat Biotech_COVAXIN	BBV152	COVID vaccine	Infectious disease	Prevention	COVID-19		NA*
CanSino_Ad5-nCoV	Convidecea/ PakVac (Pakistan)	COVID vaccine	Infectious disease	Prevention	COVID-19		NA*
CureVac_CVnCoV	No commercial name	COVID vaccine	Infectious disease	Prevention	COVID-19		NA*
Gamaleya_Sputnik V	Sputnik V	COVID vaccine	Infectious disease	Prevention	COVID-19		Numerator: total number of LMICs providing the product;<br>Denominator: total number of LMICs with populations that have the condition
Janssen (J&J)_Ad26.COV2.S	Jcovden	COVID vaccine	Infectious disease	Prevention	COVID-19		Numerator: total number of LMICs providing the product;<br>Denominator: total number of LMICs with populations that have the condition
Moderna (Spikevax)	SpikeVax (EU only for now)	COVID vaccine	Infectious disease	Prevention	COVID-19		Numerator: total number of LMICs providing the product;<br>Denominator: total number of LMICs with populations that have the condition
Novavax_NVX-CoV2373	"Covavax (India) TAK-019 (Japan)"	COVID vaccine	Infectious disease	Prevention	COVID-19		Numerator: total units of product procured by LMICs;<br>Denominator: total LMIC population with the condition
Oxford-AstraZeneca _AZD1222_AZ	Vaxzevria (EU); Covishield (SII)	COVID vaccine	Infectious disease	Prevention	COVID-19		Numerator: total number of LMICs providing the product;<br>Denominator: total number of LMICs with populations that have the condition
Oxford-AstraZeneca _AZD1222_SKB	Vaxzevria (EU); Covishield (SII)	COVID vaccine	Infectious disease	Prevention	COVID-19		Numerator: total number of LMICs with policies or roll out plans supporting the product;<br>Denominator: total number of LMICs with populations that have the condition
Pfizer-BioNTech_BNT162	Comirnaty	COVID vaccine	Infectious disease	Prevention	COVID-19		Numerator: total number of LMICs providing the product;<br>Denominator: total number of LMICs with populations that have the condition
SII_Covishield	Vaxzevria (EU); Covishield (SII)	COVID vaccine	Infectious disease	Prevention	COVID-19		Numerator: total number of LMICs with policies or roll out plans supporting the product;<br>Denominator: total number of LMICs with populations that have the condition
Sinopharm_SARS-CoV-2	BBIBP-CorV	COVID vaccine	Infectious disease	Prevention	COVID-19		Numerator: total number of LMICs providing the product;<br>Denominator: total number of LMICs with populations that have the condition
Sinovac_Coronavac	CoronaVac	COVID vaccine	Infectious disease	Prevention	COVID-19		Numerator: total number of LMICs with policies or roll out plans supporting the product;

Product	Commercial name	Type	Health condition	Continuum of care	Specific diseases	Pilot[#]	Scale up data
							Denominator: total number of LMICs with populations that have the condition
Bivalent Oral Polio Vaccine (bOPV)	Polio Sabin One and Three	Non-COVID vaccine	Infectious disease	Prevention	Polio		Numerator: total LMIC population reached with the product;<br>Denominator: total LMIC population with the condition
Ervebo	Ervebo	Non-COVID vaccine	Infectious disease	Prevention	Zaire Ebolavirus		NA*
Gardasil	Gardasil (quadrivalent)	Non-COVID vaccine	Infectious disease	Prevention	HPV		Numerator: total LMIC population reached with the product;<br>Denominator: total LMIC population with the condition
Japanese Encephalitis Vaccine	Japanese Encephalitis Vaccine Live (SA14-14-2) or CD-JEVAX	Non-COVID vaccine	Infectious disease	Prevention	Japanese Encephalitis		NA*
MenAfriVac	MenAfriVac	Non-COVID vaccine	Infectious disease	Prevention	Meningitis-A		Numerator: total LMIC population reached with the product;<br>Denominator: total LMIC population with the condition
Novel Oral Polio Vaccine type 2 (nOPV2)	Novel oral polio vaccine type 2 (nOPV2)	Non-COVID vaccine	Infectious disease	Prevention	Polio		NA*
Rotavac	Rotavac	Non-COVID vaccine	Infectious disease	Prevention	Rotavirus		Numerator: total LMIC population reached with the product;<br>Denominator: total LMIC population with the condition
RotaTeq	RotaTeq	Non-COVID vaccine	MNCH	Prevention	Rotavirus		Numerator: total LMIC population reached with the product;<br>Denominator: total LMIC population with the condition
Antenatal corticosteroids	Bexamethason brand name = Celestone Soluspan	Drug	MNCH	Prevention	Prenatal lung development		Numerator: total LMIC population reached with the product;<br>Denominator: total LMIC population with the condition
Chlorhexidine	Chlorhexidine	Drug	MNCH	Prevention	Neonatal sepsis	√	NA*
Magnesium sulfate	Magnesium sulfate, Epsom salt	Drug	MNCH	Treatment	Preeclampsia		Numerator: total number of LMICs with policies or roll out plans supporting the product;<br>Denominator: total number of LMICs with populations that have the condition
Oral rehydration solution (ORS)	Oral rehydration solution, oral rehydration salts, oral rehydration therapy, other commercial names vary extensively across countries	Drug	MNCH	Treatment	Diarrhea		Numerator: total LMIC population reached with the product;<br>Denominator: total LMIC population with the condition
Pediatric TB medicines	Rifampicin, isoniazid, pyrazinamide, ethambutol	Drug	MNCH	Treatment	TB		Numerator: total units of product procured by LMICs;<br>Denominator: total LMIC population with the condition

Product	Commercial name	Type	Health condition	Continuum of care	Specific diseases	Pilot#	Scale up data
Tranexamic acid	Cyklokapron (Pfizer)	Drug	MNCH	Treatment	Postpartum hemorrhage		NA*
Vitamin A	Vitamin A, retinol, aquasol A, retinyl acetate, retinyl palmitate	Drug/Supplementation	MNCH/Nutrition	Prevention	Nutritional deficiency	√	Numerator: total number of LMICs providing the product; Denominator: total number of LMICs with populations that have the condition
Artesunate injection	artesunate powder for injection	Drug	Infectious disease	Treatment	Malaria		Numerator: total LMIC population reached with the product; Denominator: total LMIC population with the condition
Rectal artesunate (RAS)	Rectal artesunate/artesunate suppository	Drug	Infectious disease	Treatment	Malaria		NA*
Coartem	Riamet ¬Æ, Coartem	Drug	Infectious disease	Treatment	Malaria		NA*
Coartem Dispersible	Riamet ¬Æ dispersible tablets, Coartem ¬Æ dispersible tablets (public sector)	Drug	Infectious disease	Treatment	Malaria		Numerator: total number of LMICs providing the product; Denominator: total number of LMICs with populations that have the condition
Dapivirine vaginal ring (DPV-VR)	Dapivirine Vaginal Ring, DAP, DPV, DVR, DVR-004, Ring-004, TMC-120, dapivirine IVR, dapivirine intravaginal ring, DPV-VR	Drug	Infectious disease	Prevention	HIV		NA*
Pre-exposure prophylaxis (PrEP)	Truvada	Drug	Infectious disease	Prevention	HIV	√	Numerator: total LMIC population reached with the product; Denominator: total LMIC population with the condition
Pretomanid	Pretomanid	Drug	Infectious disease	Treatment	TB		NA*
Pyramax	Pyramax	Drug	Infectious disease	Treatment	Malaria		NA*
Pyramax Granules	Pyramax Granules	Drug	Infectious disease	Treatment	Malaria		NA*
Tafenoquine	Krintafel (US)	Drug	Infectious disease	Treatment	Malaria	√	NA*
Fexinidazole	Fexinidazole tablet	Drug	NTD	Treatment	African trypanosomiasis		Scale data not available or 20% uptake not yet reached
Bilichek	BiliChek System	Device	MNCH	Diagnosis	Jaundice		NA*
Bubble CPAP	Pumani	Device	MNCH	Treatment	Prenatal lung development		NA*
ESM-Uterine Balloon Tamponade	The Every Second Matters for Mothers-Uterine Balloon Tamponade (ESM-UBT)	Device	MNCH	Treatment	Postpartum hemorrhage		NA*
MiracleFeet Brace	MiracleFeet Brace	Device	MNCH	Treatment	Club foot	√	Numerator: total units of product procured by LMICs; Denominator: total LMIC population with the condition
Moyo Fetal Heart Rate Monitor	Moyo Fetal Heart Rate Monitor	Device	MNCH	Screening			NA*
Sayana Press	DMPA-SC; Sayana Press with Uniject	Device	MNCH	Prevention	Contraception	√	Numerator: total number of LMICs providing the product; Denominator: total number of LMICs with populations that have the condition
Pratt Pouch	Pratt pouch	Device	Infectious disease	Treatment	HIV	√	NA*

Product	Commercial name	Type	Health condition	Continuum of care	Specific diseases	Pilot[#]	Scale up data
Tiny Targets	Tiny Targets	Device	NTD	Prevention	Human African trypanosomiasis	√	NA*
Bioline Malaria	Bioline Malaria Ag P.f (changed from: SD Bioline Malaria Ag P.f)	Diagnostic	Infectious disease	Diagnosis	Malaria		NA*
HIV self-test	OraQuick	Diagnostic	Infectious disease	Prevention	HIV	√	NA*
Oraquick HIV Self-Test	OraQuick HIV Self-Test	Diagnostic	Infectious disease	Diagnosis	HIV		NA*
SD Bioline Duo Rapid test	SD BiolineHIV/Syphilis Duo	Diagnostic	Infectious disease	Diagnosis	HIV and syphilis		NA*
Xpert HIV-1 Assay	Xpert ¬Æ HIV-1 Qual Assay	Diagnostic	Infectious disease	Diagnosis	HIV		NA*
Xpert MTB/RIF	Xpert MTB/RIF Assay	Diagnostic	Infectious disease	Diagnosis	TB	√	Numerator: total units of product procured by LMICs;<br>Denominator: total LMIC population with the condition
Congo Red Dot Paper Test	Congo Red Dot (CRD) Paper Test	Diagnostic	MNCH	Diagnosis	Preeclampsia		NA*
Cielo ULV	Cielo ULV Adulticide Space Spray	Vector control	Infectious disease	Prevention	Malaria		NA*
long-lasting insecticidal nets (LLINs)	Olyset Net	Vector control	Infectious disease	Prevention	Malaria	√	Numerator: total units of product procured by LMICs;<br>Denominator: total LMIC population with the condition
Royal Sentry 2.0	Royal Sentry 2.0	Vector control	Infectious disease	Prevention	Malaria		NA*
SumiShield	SumiShield 50WG	Vector control	Infectious disease	Prevention	Malaria		NA*
Tsara	Tsara Soft	Vector control	Infectious disease	Prevention	Malaria		NA*

*NA: Scale data not available or 20% uptake not yet reached

we conducted pilot data collection on 12 product and Pocket Colposcope (a screening device for cervical cancer) was excluded from final analysis due to lack of reliable data.

For more information about products, please refer to our website at: https://launchandscalefaster.org/global-milestones

Appendix 3: Missing data

Table S3. Patterns of missing milestone data

	Number of missing datapoints to which median by product type was applied (Total sample: 59)	Number of missing datapoints to which median by health condition was applied (Total sample: 59)
**LAUNCH: Ideation to first country launch**	16	0
Ideation to proof of concept	2	0
Proof of concept to first regulatory approval	17	5*
First regulatory approval to first country launch	26	5*
**SCALE: First country launch to global uptake at least 20%**	37	0
**TOTAL Ideation to global uptake at least 20%**	35	0

*All five vector control products included in this study missed the dates for proof of concept, first regulatory approval or first country launch. Since vector control products are all used for infectious diseases, we use the median of infectious diseases to estimate the missing values.

[#] Product type was selected to estimate missing values because it has better data quality than other indicators, and prior studies fund different types of products have noticeable differences in their R&D, launch and scale pathway.

For missing characteristic data, we applied the following adjustments:

- Buying environment (3 missing data points)
  - Used main market type as a proxy for buying environment (i.e. global markets are usually associated with centralized buying environments). We applied this logic to Dapivirine and Novavax_NVX-CoV2373.
  - Used product type as a proxy for buying environment (i.e. almost all vector control products have a centralized buying environment). We applied this logic to Cielo ULV.

- Behavior change (3 missing data points)
  - Assumed no behavior change required for Pretomanid, Ervebo, and Oxford-AstraZeneca_AZD1222.

- Market type (2 missing data points)
  - All vector control and covid vaccines have a global market, so we applied global market to Cielo ULV and Ervebo.

- LMIC specific (2 missing data points)
  - Assumed Pretomanid and Ervebo are LMIC specific.

Appendix 4: Descriptive statistics of original data (i.e. with missing values)

In this section, we report the descriptive analysis on the original data, in accompany with the analysis with the data after amputation in the main text. Specifically, Table S4-1 compares the product characteristics between original dataset and data after amputation. Table S4-2 presents the time between different milestones reported by the original dataset, providing additional information to supplement the Table 1 in main text. Table S4-3 presents the time between key milestones, disaggregated by characteristic to supplement the Table 2 in the main text.

Table S4-1. Product characteristics

Characteristic	Original data with missing values N*(%)	Data after amputation (N=59) N (%)
**Type of care**		
Prevention	34(58%)	34(58%)
Diagnosis or screening	8(14%)	8(14%)
Treatment	17(29%)	17(29%)
**Developer**		
Private company	32 (58%)	32 (58%)
Public/private collaboration	17 (31%)	17 (31%)
Academic/NGO	6 (11%)	6 (11%)
**Champion**		
Clear champion	28 (60%)	33 (56%)
No clear champion	19 (40%)	26 (44%)
**Buying environment**		
Centralized	26 (57%)	29 (52%)
Decentralized	20 (44%)	27 (48%)
**Country specific**		
LMIC specific	23 (39%)	23 (39%)
Not LMIC specific	36 (61%)	36 (61%)
**Product competition**		
No significant product competition	29 (59%)	34 (58%)
Significant product competition	20 (41%)	25 (42%)
**Targeting**		
Requires targeting	30 (53%)	32 (54%)
Does not require targeting	27 (47)	27 (46%)
**Main market type**		
Consumer/institutional	27 (53%)	31 (54%)
Global	24 (47%)	26 (46%)
**Target channel**		
Mixed or private	11 (23%)	20 (34%)
Public	36 (77%)	39 (66%)

* sample size varied based on number of missing values of each characteristic

Table S4-2. Time between milestones (in years)

Milestones (n)	Median	25th Percentile	75th Percentile	IQR
**LAUNCH: Ideation to first country launch (n=43)**	6.7	0.94	14.2	13.2
Ideation to proof of concept	6.9	1.4	16.1	14.7
Proof of concept to first regulatory approval	0.86	-0.06	4.3	4.3
First regulatory approval to first country launch	0.14	0.04	0.75	0.71
**SCALE: First country launch to global uptake at least 20% (n=22)**	3.4	0.72	9.3	8.5
**TOTAL Ideation to global uptake at least 20% (n=24)**	12.9	3.3	31.8	28.4

Table S4-3. Time between key milestones (in years) by characteristics

Median (IQR)		LAUNCH: ideation to first country launch	SCALE: first country launch to at least 20% global uptake
**Type of care**	**Prevention**	5.4 (14.0)	1.3 (7.8)
	**Diagnosis or screening**	3.0(10.6)	3.7(0)
	**Treatment**	6.8(26.9)	13.0(24.6)
**Type of developer**	**Private company**	4.5 (11.6)	3.4(6.0)
	**Public/ private collaboration**	9.2 (16.1)	0.6 (21.6)
	**Academic/ NGO**	4.0 (3.1)	13.0 (26.5)
**Champion**	**Clear champion**	4.5 (11.6)	2.3 (4.6)
	**No clear champion**	6.7 (17.8)	9.9 (22.1)
**Buying environment**	**Centralized**	9.4 (13.5)	4.1 (6.8)
	**Decentralized**	0.95 (5.4)	1.3 (17.4)
**Country specific**	**LMIC specific**	4.2 (6.0)	5.0 (13.1)
	**Not LMIC specific**	7.9 (18.1)	3.0 (9.3)
**Product competition**	**No significant competition**	3.6 (8.7)	1.6 (4.8)
	**Significant competition**	12.0 (15.9)	5.0 (17.9)
**Targeting**	**Required**	2.4 (13.2)	1.6 (7.3)
	**Not required**	9.3 (11.0)	5.7 (20.7)
**Main market type**	**Consumer/ institutional**	1.8 (6.2)	1.6 (4.6)
	**Global**	13.0 (18.3)	5.0 (10.2)
**Target channel**	**Mixed or private**	6.8 (10.3)	4.7 (10.9)
	**Public**	4.2 (15.7)	1.3(7.8)

Appendix 5: Alternative regression models

This section presents the additional regression analysis in accompany with the main models in the manuscript (Table 3). The dependent variables and independent variables remain the same as those in main models. Specifically, Table S5-1 used the same model setting, robustness regression, as those in the main models, but on a subset of data without COVID-19 products. Results have showed that most factors that are positively or negatively associated with the launch and scale timelines remain the same as those in main models.
Table S5-1 presents the results from OLS models, as the robustness check for the main models. Table S5-3 presents robustness regression analysis on the original data. Since we filled the data gaps based on assumptions, the analysis on original data presents findings without the potential bias that might be the results of data amputation approach. However the results remain comparable to the main models, which further indicated the data amputation process was conducted properly. Table S5-4&5 explored the different combination of independent variables, adding additional rigor to the main model.

Table S5-1. Robustness regression without COVID-19 vaccines

**Independent Variables (Categorical)**		**Model 3: LAUNCH (ideation to first country launch)**	**Model 4: SCALE (first country launch to global uptake at least 20%)**
**(reference group)**		Beta (p value) [90%CI]	Beta (p value) [90%CI]
Health condition (Non-COVID-19 IDs and NTDs)	MCH (and nutrition)	2.49(0.41)	-0.44(0.89)
		[-2.52,7.50]	[-5.50,4.63]
	COVID-19	NA	NA
		NA	NA
Type of care (Prevention)	Diagnosis or screening	-2.11(0.54)	-9.28**(0.01)
		[-7.86,3.65]	[-15.10, -3.45]
	Treatment	-3.67 (0.18)	7.46***(<0.01)
		[-8.23]	[2.84,12.07]
Type of developer (Private company)	Academic or NGOs	6.11*(0.08)	-4.13(0.24)
		[0.33,11.89]	[-9.98,1.71]
	Public-private collaboration	6.63**(0.03)	2.47(0.40)
		[1.80,11.45]	[-2.41,7.36]
Champion (Clear champion)	No clear champion	-0.039(0.99)	-0.96(0.67)
		[-3.77,3.69]	[-4.73,2.81]
Buying environment (Decentralized)	Centralized	3.10(0.28)	2.46(0.40)
		[-1.68,7.89]	[-2.38,7.30]
Country specific (Not LMIC specific)	LMIC Specific	-6.75***(<0.01)	-6.07***(<0.01)
		[-10.36, -3.14]	[-9.72, -2.42]
Product competition (No significant product competition)	Significant product competition	1.85(0.40)	-0.68(0.76)
		[-1.82,5.52]	[-4.39,3.04]
Targeting (Requires targeting)	Does not require targeting	-1.84(0.44)	4.51*(0.07)
		[-5.81,2.14]	[0.49,8.53]
Main market type (Global market)	Consumer or institutional market	-6.80**(0.02)	-0.88(0.76)
		[11.48, -2.13]	[-5.61,3.85]
Target channel (Public)	Private or mixed	0.011(0.99)	4.51*(0.10)
		[-4.40,4.43]	[0.04,8.97]
Constant	Constant	14.02	7.88
Model metrics	Observations	46	46

*** p<0.01, ** p<0.05, * p<0.1

Table S5-2. Regression results using OLS models

Independent Variables (Categorical)		Model 5: LAUNCH (ideation to first country launch)	Model 6: SCALE (first country launch to global uptake at least 20%)
**(reference group)**		Beta (p value) [90%CI]	Beta (p value) [90%CI]
Health condition (Non-COVID-19 IDs and NTDs)	MCH (and nutrition)	-2.90(0.36)	1.28(0.69)
		[-8.12,2.33]	[-4.06,6.62]
	COVID-19	-17.36***(<0.01)	-11.72***(<0.01)
		[-23.12, -11.60]	[-17.61, -5.84]
Type of care (Prevention)	Diagnosis or screening	-4.62(0.20)	-8.14**(0.03)
		[-10.53,1.31]	[-14.19, -2.09]
	Treatment	-1.63(0.56)	5.49*(0.06)
		[-6.33,3.07]	[0.69,10.30]
Type of developer (Private company)	Academic or NGOs	7.68**(0.04)	0.17(0.96)
		[1.65,13.70]	[-5.99,6.33]
	Public-private collaboration	3.99(0.11)	2.74(0.28)
		[-0.09,8.08]	[-1.43,6.92]
Champion (Clear champion)	No clear champion	-2.66(0.25)	-3.14(0.19)
		[-6.51,1.20]	[-7.09,0.80]
Buying environment (Decentralized)	Centralized	5.78**(0.04)	3.91(0.18)
		[1.09,10.47]	[-0.88,7.70]
Country specific (Not LMIC specific)	LMIC Specific	-8.72***(<0.01)	-6.31***(<0.01)
		[-12.23, -5.21]	[-9.89, -2.72]
Product competition (No significant product competition)	Significant product competition	1.41(0.53)	1.69(0.46)
		[-2.32,5.15]	[-2.12,5.51]
Targeting (Requires targeting)	Does not require targeting	0.263(0.91)	1.49(0.53)
		[-3.58,4.10]	[-2.43,5.51]
Main market type (Global market)	Consumer or institutional market	-5.856**(0.05)	-3.71(0.21)
		[-10.66, -1.05]	[-8.62,1.20]
Target channel (Public)	Private or mixed	-1.448(0.57)	0.89(0.73)
		[-5.67,2.77]	[-3.43,5.20]
Constant	Constant	18.45	11.84
Model metrics	Observations	59	59
	R-squared	0.59	0.56

*** $p<0.01$, ** $p<0.05$, * $p<0.1$

Table S5-3. Robustness regression results with original data

Independent Variables (Categorical)		Model 7: LAUNCH (ideation to first country launch)
(reference group)		Beta (p value) [90%CI]
Health condition (Non-COVID-19 IDs and NTDs)	MCH (and nutrition)	-4.68(0.10)
		[-9.37,0.02]
	COVID-19	-11.47***(<0.01)
		[-16.62, -6.32]
Type of care (Prevention)	Diagnosis or screening	-7.90**(0.05)
		[-14.44, -1.35]
	Treatment	-2.23(0.51)
		[-7.87,3.40]
Type of developer (Private company)	Academic or NGOs	3.05(0.38)
		[-2.80,8.91]
	Public-private collaboration	2.11(0.32)
		[-1.40,5.62]
Champion (Clear champion)	No clear champion	1.09(0.62)
		[-2.61,4.79]
Buying environment (Decentralized)	Centralized	2.96(0.30)
		[-1.77,7.69]
Country specific (Not LMIC specific)	LMIC Specific	-5.12***(<0.01)
		[-8.19, -2.04]
Product competition (No significant product competition)	Significant product competition	10.54***(<0.01)
		[6.39,14.68]
Targeting (Requires targeting)	Does not require targeting	-3.54*(0.09)
		[-7.05, -0.02]
Main market type (Global market)	Consumer or institutional market	-5.69**(0.03)
		[-10.04, -1.34]
Target channel (Public)	Private or mixed	-0.029(0.99)
		[-4.15,4.09]
Constant	Constant	15.11
Model metrics	Observations	43

*** p<0.01, ** p<0.05, * p<0.1

Model on first country launch to global uptake at least 20% is not provided here, as severe collinearity has been detected (VIF >5), mainly due to the small sample size of original data (N=25)

Table S5-4. Robust regression models with alternative dependent variables: from ideation to first country launch

Independent Variables (Categorical) (reference)	Variable	M8	M9	M10	M11	M12	M13	M14	M15	M16	M17	M18	M19
		Beta(SE)	Beta(SE)	Beta(SE)	Beta(SE)	Beta(SE)	Beta(SE)	Beta(SE)	Beta(SE)	Beta(SE)	Beta(SE)	Beta(SE)	Beta(SE)
Health condition (Non-COVID-19 IDs and NTDs)	MCH (and nutrition)	-0.89	0.79	-0.94	-1.99	-4.62***	-1.43	-1.31	-0.84	1.69	-1.28	1.96	-0.39
		(2.05)	(2.44)	(2.13)	(2.27)	(1.41)	(1.56)	(2.21)	(2.09)	(2.23)	(2.19)	(1.88)	(2.87)
	COVID-19	-10.90***	-11.12***	-11.23***	-13.40***	-11.13***	-11.45***	-10.92***	-11.02***	-7.74***	-10.82***	-9.21***	-11.71***
		(2.32)	(2.32)	(2.50)	(2.91)	(1.71)	(1.81)	(2.64)	(2.40)	(2.76)	(2.35)	(2.30)	(3.15)
Type of care (Prevention)	Diagnosis or screening	-2.01	-1.65	-2.21	-3.67	-0.16	-0.83	-3.24	-1.85	-1.08	-2.32	-1.34	-3.73
		(2.69)	(2.65)	(2.81)	(2.94)	(1.93)	(2.03)	(2.86)	(2.89)	(2.71)	(2.88)	(2.25)	(3.26)
	Treatment	-0.45	-0.63	-0.47	-1.22	1.96	0.56	-0.83	-0.49	0.10	-0.61	-0.09	-3.46
		(2.13)	(2.29)	(2.25)	(2.26)	(1.47)	(1.61)	(2.30)	(2.20)	(2.11)	(2.18)	(1.75)	(2.57)
Type of developer (Private company)	Academic or NGOs		-0.71										2.89
			(2.90)										(3.242)
	Public -private collaboration		3.39										4.59**
			(2.07)										(2.25)
Champion (Clear champion)	No clear champion			-0.25									-0.64
				(1.94)									(2.13)
Buying environment (Decentralized)	Centralized				2.31								4.44*
					(2.15)								(2.58)
Country specific (Not LMIC specific)	LMIC Specific						-5.10***					-5.44***	
							(1.36)					(1.49)	
Product competition (No significant product competition)	Significant product competition							2.29					3.99*
								(1.99)					(2.05)
Targeting (Requires targeting)	Does not require targeting								-0.37				-2.10
									(1.92)				(2.12)
Main market type (Global market)	Consumer or institutional market									-3.81*		-3.88**	-4.33
										(2.08)		(1.74)	(2.63)
Target channel (Public)	Private or mixed										0.82		1.69
											(2.12)		(2.32)
Constant	Constant	11.80	10.46	12.16	12.34	12.03	13.06	11.65	11.98	12.17	11.66	14.45	10.69
Model metrics	Observations	59	59	59	59	59	59	59	59	59	59	59	59

Table S5-5. Robust regression models with alternative dependent variables: from first country launch to global uptake at least 20%

Independent Variables (Categorical) (reference)	Variable	M20	M21	M22	M23	M24	M25	M26	M27	M28	M29	M30	M31
		Beta(SE)	Beta(SE)	Beta(SE)	Beta(SE)	Beta(SE)	Beta(SE)	Beta(SE)	Beta(SE)	Beta(SE)	Beta(SE)	Beta(SE)	Beta(SE)
Health condition (Non-COVID-19 IDs and NTDs)	MCH (and nutrition)	-0.30*	-0.51**	-0.32*	-0.31*	-0.23	-0.40**	-0.28	-0.29*	-0.40**	-0.24	-0.38	1.63***
		(0.16)	(0.20)	(0.16)	(0.17)	(0.17)	(0.18)	(0.25)	(0.16)	(0.18)	(0.17)	(0.24)	(0.43)
	COVID-19	-4.29***	-4.34***	-4.17***	-4.31***	-6.87***	-4.15***	-5.48***	-4.29***	-4.43***	-4.27***	-4.95***	-4.53***
		(0.18)	(0.19)	(0.19)	(0.22)	(0.20)	(0.21)	(0.30)	(0.19)	(0.23)	(0.18)	(0.29)	(0.47)
Type of care (Prevention)	Diagnosis or screening	-1.35***	-1.35***	-1.29***	-1.36***	-3.95***	-1.21***	-2.11***	-1.35***	-1.39***	-1.3***	-1.72***	-2.51***
		(0.20)	(0.22)	(0.22)	(0.22)	(0.23)	(0.23)	(0.33)	(0.22)	(0.22)	(0.23)	(0.31)	(0.49)
	Treatment	16.25***	16.15***	16.31***	16.24***	13.65***	16.44***	15.44***	16.25***	16.23***	16.28***	15.88***	15.11***
		(0.16)	(0.19)	(0.17)	(0.167)	(0.17)	(0.19)	(0.26)	(0.17)	(0.17)	(0.17)	(0.25)	(0.39)
Type of developer (Private company)	Academic or NGOs		0.46*										-0.51
			(0.24)										(0.49)
	Public -private collaboration		0.09										0.28
			(0.17)										(0.34)
Champion (Clear champion)	No clear champion			0.12									-0.07
				(0.15)									(0.32)
Buying environment (Decentralized)	Centralized				0.02								0.81**
					(0.16)								(0.39)
Country specific (Not LMIC specific)	LMIC Specific						0.26*					0.25	
							(0.16)					(0.20)	
Product competition (No significant product competition)	Significant product competition							-0.71***				-0.51**	-1.04***
								(0.23)				(0.21)	(0.31)
Targeting (Requires targeting)	Does not require targeting								0.01				0.32
									(0.15)				(0.32)
Main market type (Global market)	Consumer or institutional market									0.11			-2.09***
										(0.17)			(0.40)
Target channel (Public)	Private or mixed										-0.08		-0.037
											(0.17)		(0.35)
Constant	Constant	4.84	4.83	4.71	4.85	7.43	4.63	6.04	4.84	4.85	4.83	5.47	6.30
Model metrics	Observations	59	59	59	59	59	59	59	59	59	59	59	59

Appendix 6: Timeline for scale-up (selected products)

Table S6. Products with scale up data and their scale up timeline

Product	Ideation	First country launch	LMIC uptake 20%	LMIC uptake 50%	LMIC uptake 80%
Xpert MTB/RIF	2008-10-01	2011-03-01	2015-07-01	2016-07-01	2017-07-01
Vitamin A	1947-07-01	1970-07-01	2000-07-01	2003-07-01	2005-07-01
Pediatric TB medicines	2011-03-01	2015-12-01	2017-07-01	2018-07-01	2019-07-01
Bivalent Oral Polio Vaccine	2005-10-13	2009-12-15	2016-05-02	2016-05-02	2016-05-02
LLINs	1993-07-01	2002-07-01	2007-07-01	2017-07-01	
Oral rehydration solution	1962-08-04	1969-07-01	1990-07-01	2000-07-01	
Japanese Encephalitis Vaccine	1954-07-01	1988-07-01			2020-07-01
Oraquick HIV Self-Test	2004-07-01	2017-07-01		2020-07-01	
PrEP	2008-01-15	2015-12-01	2019-10-01		
Sayana Press	1995-07-01	2014-07-10	2014-07-01		
MiracleFeet Brace	2012-07-01	2015-07-01	2020-07-01		
MenAfriVac	2001-07-01	2010-09-01	2020-07-01		
Artesunate injection	1979-12-01	1992-07-01	2021-07-01		
Rotavac	1993-11-01	2015-03-01	2020-07-01		
RotaTeq	1982-07-01	2006-10-27	2020-07-01		
Pfizer-BioNTech_BNT162	2020-01-10	2020-12-24	2021-11-29		
Janssen (J&J)_Ad26.COV2.S	2020-03-30	2021-02-17	2021-05-21		
Gamaleya_Sputnik V	2020-06-10	2020-12-29	2021-07-01		
Sinovac_Coronavac	2020-01-01	2020-10-15	2022-05-04		
Novavax_NVX-CoV2373	2020-02-26	2021-11-01	2021-05-06		
Moderna (Spikevax)	2020-01-13	2020-12-20	2021-12-10		
